# Factors affecting the foraging behaviour of the European shag: *implications for seabird tracking studies*

**DOI:** 10.1007/s00227-014-2422-x

**Published:** 2014-04-03

**Authors:** L. M. Soanes, J. P. Y. Arnould, S. G. Dodd, G. Milligan, J. A. Green

**Affiliations:** 1 School of Environmental Sciences, University of Liverpool, Liverpool, L69 3GP UK; 2 School of Life and Environmental Sciences, Deakin University, Burwood, 3215 Australia; 3 Royal Society for the Protection of Birds, North Wales Office, Bangor, LL57 4FD UK

## Abstract

Seabird tracking has become an ever more popular tool to aid environmental procedures such as the designation of marine protected areas and environmental impact assessments. However, samples used are usually small and little consideration is given to experimental design and sampling protocol. European shags *Phalacrocorax aristotelis* were tracked using GPS technology over three breeding seasons and the following foraging trip characteristics: trip duration, trip distance, maximum distance travelled from the colony, size of area used and direction travelled from colony were determined for each foraging trip. The effect of sex, year of study, breeding site, number and age of chicks and the timing of tracking on foraging behaviour were investigated using a General Estimation Equation model. A range of sampling scenarios reflecting likely field sampling were also tested to compare how foraging behaviour differed depending on composition of the sample of birds tracked. Trip distance, trip duration, maximum distance travelled and size of area used were all significantly affected by the breeding site, and the number of chicks a tracked adult was raising. The effect of sex was also seen when examining trip distance, trip duration and the maximum distance travelled. The direction travelled on a foraging trip was also significantly affected by breeding site. This study highlights the importance of sampling regime and the influence that year, sex, age, number of chicks and breeding site can have on the foraging trip characteristics for this coastal feeding seabird. Given the logistical and financial constraints in tracking large numbers of individuals, this study identifies the need for researchers to consider the composition of their study sample to ensure any identified foraging areas are as representative as possible of the whole colony’s foraging area.

## Introduction

In recent years, the number of seabird tracking studies using global positioning system data loggers, satellite transmitters and geolocators has increased substantially due to the availability of cheaper and smaller technologies, adding greatly to our understanding of seabird behaviour and ecology (Evans et al. 2013; Hazen et al. 2012; Burger and Shaffer 2008). A range of seabird species have now been tracked, ranging from the 150-g Thin-billed prion *Pachyptila belcheri* (Quillfeldt et al. 2012) to the 12-kg Wandering albatross *Diomedea exulans* (Shaffer et al. 2005; Gremillet et al. 2012). These studies have been used to inform the designation and effectiveness of marine protected areas (e.g. BirdLife 2010; Harris et al. 2007; Hyrenbach et al. 2006), to provide data for environmental impact assessments (e.g. Perrow et al. 2006; Soanes et al. 2012), to examine the effects of environmental change (e.g. Durant et al. 2009; Wanless et al. 2007; Wilson et al. 2002) and to assess changes in fishery practices (e.g. Bugoni et al. 2009; Copello and Quintana 2009) (Table 1). However, the logistical and financial constraints of working at seabird colonies often means that samples of individuals used in tracking studies may fail to properly represent the traits of the population. A review of recent literature (Table 1) revealed that seabird tracking studies used a mean sample size of 29 individuals in each year of study (range 4–124, median = 23, *n* = 33). Sample size in these studies were found to represent a mean of only 1.4 % of the total colony size (range 0.001–25 %, *n* = 30 colonies), which is somewhat biased by the study of Stenhouse et al. (2012) who tracked 30 individuals from a colony of 65 pairs. If this study is excluded from the sample, we find that the sample size used at the remaining colonies represented only 0.7 % (range 0.001–6.6 %, *n* = 29 colonies) of the study colony. Birds were tracked for more than one field season in just four out of the 22 of the studies, eight of the studies reported the sex of tracked birds, and three covered a range of breeding stages (e.g. incubating and chick-rearing individuals). Whilst the reporting of these factors may not be applicable to all studies (e.g. for wintering area distributions). These factors are likely to influence the results of tracking studies undertaken during the breeding season when only small samples are used.

**Table 1:** A summary of the published papers returned, between November 2011–2012, when the term “seabird tracking” was entered into the search engine Web of Knowledge^TM^ (Thomson Reuters, USA)

Species (scientific name)	Focus of study	Number of individuals deployed	Size of colony (pairs)	Sex of birds reported	Included range of breeding stages	Tracked in more than 1 year	References
*Fregata magnificens*	Environmental variability	16	1,200	N	N	N	De Monte et al. (2012)
*Sula variegata*	Fisheries	26	172,480	Y	N	N	Bertrand et al. (2012)
*Morus bassunus*	Sex specific foraging	36, 42, 27	40,000	Y	Y	Y	Stauss et al. (2012)
*Morus bassunus*	Renewable energy	23	4,500	N	N	N	Soanes et al. (2012)
*Morus bassunus*	Pollution risk	64	–	Y	Y	Y	Montevecchi et al. (2012)
*Morus capensis*	Foraging areas at two sites	21, 25	32,000, 84,000	N	N	N	Moseley et al. (2012)
*Phalacrocorax bougainvillii* and *Sula variegata*	Comparison between species	20, 51	240,000, 41,000	Y	N	N	Weimerskirch et al. (2012)
*Thalassarche melanophrys*	Habitat use	49	74,000	N	Y	N	Wakefield et al. (2012)
*Calonectris diomedea*	Trophic level specialisation	23, 29	30,000, 850	N	N	N	Alonso et al. (2012)
*Puffinus mauretanicus*	Breeding foraging areas	6	–	Y	N	N	Louzao et al. (2012)
*Puffinus mauretanicus*	Year round foraging areas	26	200	Y	N/A	N/A	Guilford et al. (2012)
*Calonectris diomedea*	Fisheries and climate change	100	29,540	N	N/A	N	Ramos et al. (2012)
*Rissa tridactyla*	Wintering areas at 18 sites	10–16	–	N	N/A	Y	Frederiksen et al. (2012)
*Fratercula arctica*	Renewable energy	7	40,000	N	N	N	Harris et al. (2012)
*Alle alle*	Wintering areas	124	3,500,000	N	N/A	N	Fort et al. (2012)
*Alle alle*	Breeding foraging areas	13	–	N	N	N	Jakubas et al. (2012)
*Spheniscus magellanicus*	Comparison of six colonies	7–18	2,000–32,337	N	N	N	Sala et al. (2012)
*Eudyptes chrysocome*	Comparison of three colonies	22, 20,20	50,000–150,000	Y	N/A	N	Thiebot et al. (2012)
*Xema sabini*	Migration	5	60	N	N/A	N	Stenhouse et al. (2012)
*Larus atlanticus*	Breeding foraging areas	(2 years) 10, 12	91	N	N	Y	Suarez et al. (2012)
*Ichthyaetus audouinii*	Breeding foraging areas	8	12,000	Y	N	N	Christel et al. (2012)
*Stercorarius skua*	Foraging areas	7,11,4	N/A, N/A, 2170	N	N/A	N	Magnusdottir et al. (2012)

Colony size was reported in only half of the publications, for those which did not report colony size it was found (when available) through the Birdlife International database (www.birdlife.org/datazone)

These limited sampling regimes are likely to fail to adequately represent population-level characteristics. This is due in part, to the known variability in seabird foraging behaviour due to effects such as inter-individual differences caused by underlying physiology (Biro and Stamps 2010; Sommerfeld et al. 2013), sex-related differences (Weimerskirch et al. 2009; Pinet et al. 2012), age and experience (Daunt et al. 2007), environmental factors (Chivers et al. 2012), location of breeding site (Hipfner et al. 2007), stage of breeding clutch size and size of colony (Wakefield et al. 2013). Soanes et al. (2013) highlight the need for researchers to explore and accept the limitations of their data sets before drawing conclusions on the location and extent of a whole colony’s important foraging areas by considering the number of individuals and foraging trips included in a sample. Therefore, whilst it may not be possible to sample a large number of individuals from any particular colony, we should ensure that the individuals that are sampled are as representative as possible of the whole study population.

In this study, we examined the effect of a range of factors on the foraging behaviour of European shags *Phalacrocorax aristotelis* breeding at Puffin Island, Wales, Great Britain a designated special protected area (SPA). The European shag is a good model species for testing these interactions given that they are a dimorphic species and can easily be sexed by their calls (Snow 1963). During breeding, they exhibit variability in the number of eggs laid per female (ranging from 1 to 4 eggs) and in chick-rearing success with not all chicks surviving to fledging. Furthermore, the distribution of breeding sites at this study colony can be classified into separate “sub-colonies” and 3 years worth of tracking data was collected from this species at this site allowing the effect of year on foraging behaviour to be investigated. The European shag is considered an Amber listed species in Europe (Eaton et al. 2009) and at 494 breeding pairs the Puffin Island colony is the largest population in Wales (Goddard 2010). This species has also been identified as having good potential for acting as a reliable ecological indicator on the state of the marine environment (Fortin et al. 2013). The effects of year, sex, age of chicks, timing of tracking, number of chicks being raised and the breeding site on foraging trip distance, duration, maximum distance travelled from the colony and the size of the area used were tested with the aim of determining which, if any, are the most important factors to consider when planning and undertaking a seabird tracking study. We then simulated different realistic sampling regimes to evaluate how sample selection can influence conclusions on apparent foraging characteristics.

## Methods

### Field methods

European Shags (from here on referred to as “shags”), breeding on Puffin Island, Great Britain (53.3°N, 4.0°W) were tracked using IgotU GT-120 GPS data loggers (Mobile Action, Taiwan) over three consecutive breeding seasons (2010–2012). Birds were caught whilst brooding chicks at their nests using a crooked pole. A total of 28, 31 and 25 individuals were instrumented in 2010, 2011 and 2012, respectively, 11 individuals were tracked in more than 1 year. Loggers were deployed between the 9th May and 18th June of each year (which represents the main chick-rearing period for this species) and samples represented males and females, individuals breeding at three different sites, with different numbers of chicks at the time of tracking, and with varying ages of chicks (from 1 to 35 days) (Table 2). Loggers were attached to the back feathers with waterproof Tesa^®^ Extra power tape (Wilson et al. 1997) and weighed 15 g when packaged, which equates to <1 % of a shags body weight. The battery life of these loggers was 5–6 days when set to record a location every 2 min.

**Table 2:** Sample sizes used for each explanatory variable included in the General Estimation Equation model

	Sample size	Number of foraging trips
*Sex*		
Males	28	302
Females	29	261
*Site*		
Ledge	32	293
North side	17	197
Beach	8	73
*Year*		
2010	20	174
2011	16	161
2012	21	228
*Number of chicks*		
1 Chick	7	59
2 Chicks	20	191
3 Chicks	30	313
Total	57	563

Regular visits to nests before, during and after tracking allowed us to estimate the age of the chicks and allowed us to monitor the productivity of the nests which were compared to control nests each year to assess any detrimental effects of tracking. The number of chicks that reached approximately 30–35 days old per nest was recorded (this is the age when they became mobile and were difficult to assign to individual nests) as an estimate of the productivity of each nest. A Kruskal–Wallis one-way analysis was conducted on this productivity data to assess site differences.

### Tracking data

The GPS devices did not always record a position every 120 s as programmed to do so, in part due to the diving activity of shags. This may provide a biased sample of the spatial distribution of foraging activity (McLeay et al. 2010), and so GPS fixes were interpolated to every 10 s using the software R (R Development Core Team 2011) with the package “trip” (Sumner 2012). This package was also used to calculate the area covered on each foraging trip by calculating the time spent in a pre-defined grid of 1 × 1 km cells surrounding the breeding colony. The number of cells used on each trip was used to represent the size of the area (km^2^) covered on each foraging trip. Total trip distance (km), trip duration (min), the maximum distance travelled from the colony (km) and the direction travelled were also calculated for each trip.

### Statistical methods

We tested the effects of a range of categorical and continuous explanatory variables including: (1) sex of the bird, (2) number of chicks, (3) age of chicks at the time of tracking, (4) location of nest on the island, (5) date that tracking was undertaken and (6) year of tracking on the five foraging trip response variables described above. Our aim was to determine which, if any, might account for the variation in foraging behaviour that was observed between individuals. Total trip distance, trip duration and the maximum trip distance were *ln*-transformed. Generalised estimation equations (GEEs) (Liang and Zeger 1986) were used in the analysis; this allowed for compound correlation structures to be specified for each individual, in order to account for within-individual correlation. They also are more suitable than the more commonly used General Linear Models for understanding population effects rather than individual-specific effects. The models were implemented in the “geepack” version 1.1-6 package (Højsgaard et al. 2012) in the R software environment (R Development Core Team 2011). All models incorporated the same terms consisting of sex, number of chicks, year of study and location of breeding site as fixed factors and the numeric factors of age of chicks at time of tracking and the number of days into the tracking season that tracking was undertaken (days from 1st April of each year). The model outputs were analysed using one-way ANOVAs, and significant terms at *p* < 0.05 level were then submitted to post hoc Tukey comparison tests to ascertain within-factor differences. In addition to the main model, the circular statistic software Oriana (Kovach Computing Services, UK) for windows and the Watson–Williams *F* test (Batschelet 1981) were also used to analyse any differences in the direction travelled in relation to the explanatory variables. In all analyses, a significance level of *p* < 0.05 was applied.

Maps of time spent in pre-defined grid cells of 1 × 1 km were plotted to compare use of space by shags around the colony for the explanatory variables that were found to be significant after the GEE model was run (those with a *p* value <0.05). Home-range areas were represented as the actual time spent in a pre-defined grid of 1 × 1 km cells surrounding the breeding colony (Page et al. 2006). The 1 × 1 km cells that the animals spent 100 % of their time was used to represent their area of active use and the cells that the animals spent 50 % of their time (after ranking for frequency of use was) used to represent their core-foraging areas (Casper et al. 2010; Soanes et al. 2013).

A range of sampling scenarios to represent commonly implemented field sampling campaigns was also simulated and compared. Likely scenarios were selected by reviewing the literature for tracking studies of the European shag and other closely related species. For example, Cook et al. (2007) tracked samples of 8, 5 and 16 chick-rearing Cape cormorants *Phalacrocorax capensis* (from three colonies) representing both sexes over a 7-week period, Quintana et al. (2011) tracked 27 male and 26 female Imperial cormorants *Phalacrocorax atriceps* over three breeding seasons but only during the first 2 weeks of chick-rearing, Kotzerka et al. (2011) tracked 14 chick-rearing male Pelagic cormorant *Phalacrocorax pelagicus* over a 6-week period and Watanabe et al. (2011) tracked 26 (20 males and six female) Kerguelen cormorants *Phalacrocorax verrucosus* which were rearing one or two chicks only. Eight sampling scenarios were devised; (A1) shags tracked between the 1–14 May 2010 versus (A2) shags tracked between the 15 May–14 June 2010; (B1) shags breeding at the ledge site in 2010 versus (B2) shags breeding at the North side and beach sites in 2010; (C1) shags with chicks under 14 days old in 2011 versus (C2) shags with chicks over 14 days old in 2011 and finally (D1) all shags tracked in 2011 compared with (D2) all shags tracked in 2012. These samples included 6–11 individuals (29–181 foraging trips) reflecting commonly used field sample sizes. Differences between the sampling scenarios were tested using a two-sample *t* test, (significance level of *p* < 0.05).

## Results

**Fig. 1:**
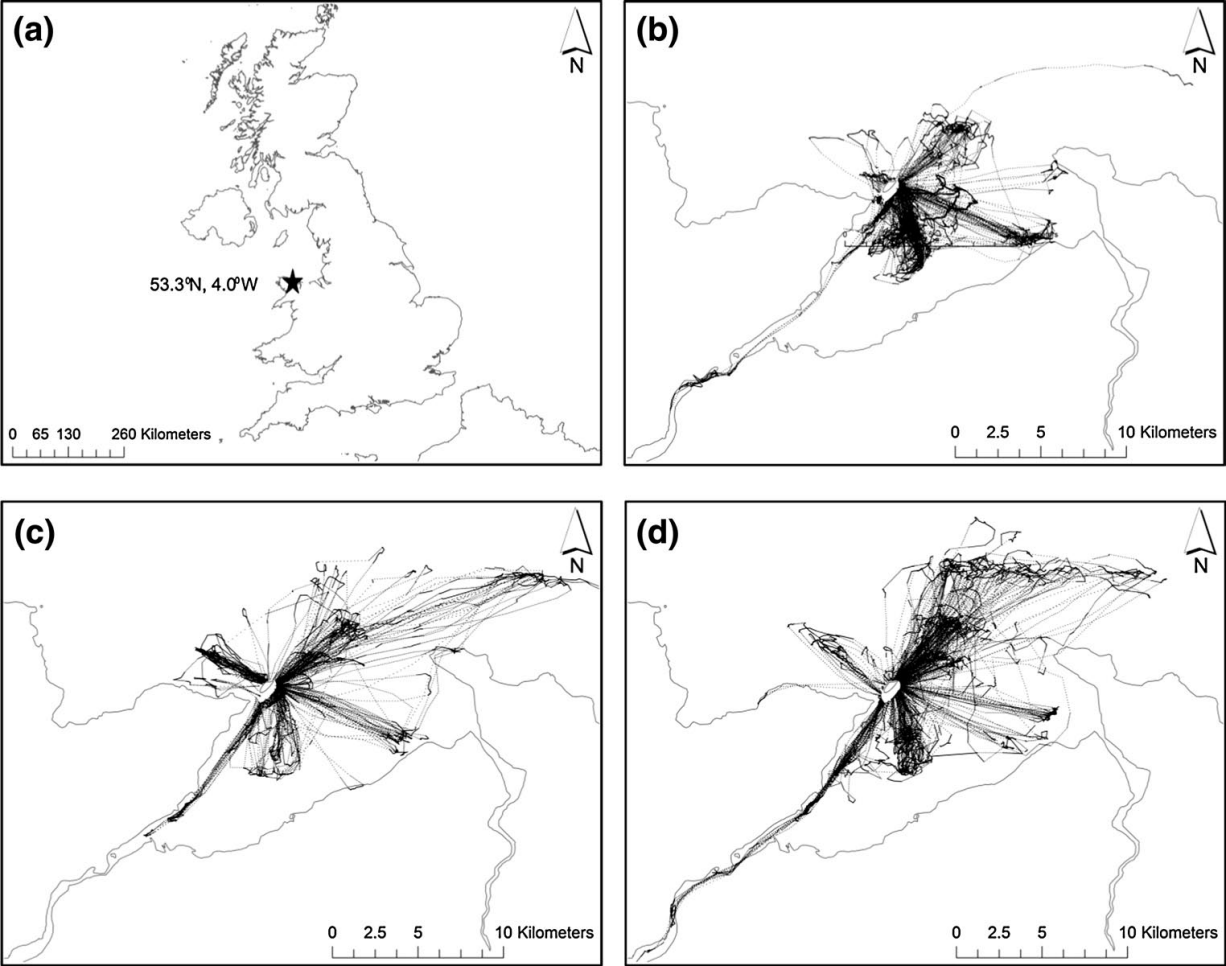
Maps showing **a** location of Puffin Island, Great Britain and the foraging trips recorded from European shags breeding in **b** 2010 **c** 2011 and **d** 2012

Each year, 18–21 loggers (20 in 2010, 18 in 2011 and 21 in 2012) were retrieved from shags breeding on Puffin Island. Two loggers in 2011 were retrieved waterlogged and all others were lost by the birds before they could be recaptured. Data from a mean of 9.7 (±0.6 SEM) foraging trips were obtained per individual (range 2–20 trips). Total foraging trip distance ranged from 0.5 to 58 km, and birds travelled 0.3–30 km from the colony. Trip duration was between 10 and 439 min. All foraging trips recorded over all years are shown in Fig. 1.

Productivity of study nests was recorded as 1.8, 1.5, and 2.6 chicks per nest in 2010, 2011 and 2012, respectively. This compared favourably to 1.2, 1.4 and 1.8 chicks per nest recorded in control nests in each year. The number of chicks reaching 30–35 days old in each nest in our study was compared between each breeding site using a Kruskal–Wallis one-way analysis on ranks. No significant differences were found in the number of chicks raised between the sites (*H*_*df*=2_ = 2.971, *p* = 0.226).

### Trip distance

**Fig. 2:**
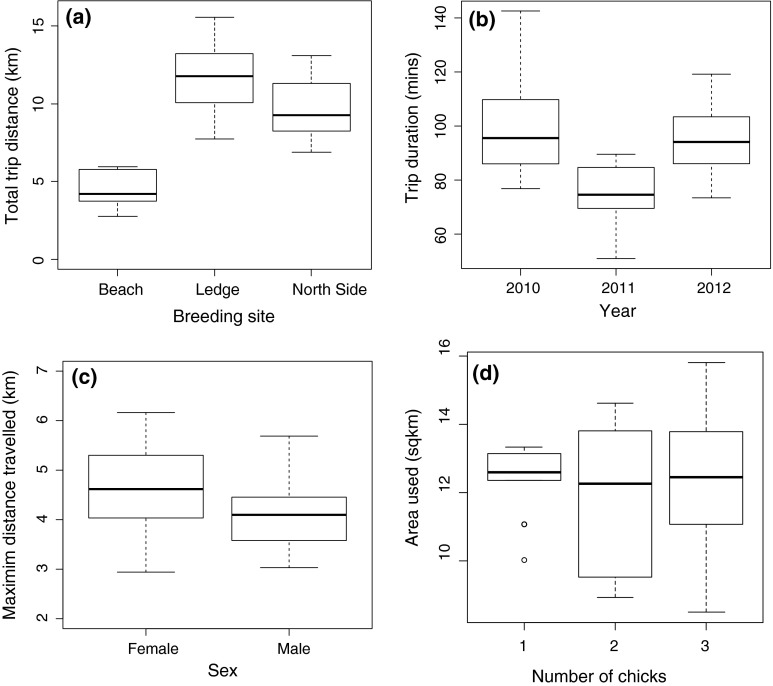
Examples of the mean differences in the foraging trip response variables significantly affected by breeding site, year of study, sex and the number of chicks an individual was raising

Total trip distance was found to differ significantly between the sexes (*F* = 13.59, *df* = 1, *p* < 0.001), with males travelling shorter distances (mean 8.4 ± 0.5, range 0.5–40 km) than females (mean 11.1 ± 0.5, range 0.9–58 km). Significant differences (*F* = 4.8, *df* = 2, *p* = 0.022) were also observed in total trip distance between individuals raising one chick (mean 9.0 ± 0.8, range 0.9–28 km) and individuals raising three chicks (mean 10.7 ± 0.5, range 0.8–58 km) (Table 3). Significant differences between all breeding sites were also observed (*F* = 34.2, *df* = 2, *p* < 0.05) with shags breeding at the ledge site, exhibiting the greatest trip distance (mean 11.6 ± 0.6, range 0.8–58 km) compared with those breeding at the North site (mean 9.4 ± 0.6, range 1–48 km) and those breeding at the beach site (mean 4.4 ± 0.6, range 0.5–49 km). The timing of tracking during the breeding season also had a significant effect on foraging trip distance (*p* = 0.03) (Fig. 2).

**Table 3:** Summary of mean estimates (±SEM) by response variable and explanatory factor

	Trip distance			Trip duration			Maximum distance			Area used		
	Mean	Contrast	*p* value	Mean	Contrast	*p* value	Mean	Contrast	*p* value	Mean	Contrast	*p* value
*Sex*												
Female	11.1 ± 0.5	F:M	**<0.001**	94.6 ± 4.6	F:M	**0.04**	4.6 ± 0.2	F:M	**0.001**	12.6 ± 0.60	F:M	0.09
Male	8.4 ± 0.5			75.2 ± 2.9			3.6 ± 0.2			11.3 ± 0.5		
*Chicks*												
One	9.0 ± 0.8	1:2	0.44	75.2 ± 5.1	1:2	0.10	3.7 ± 0.3	1:2	0.323	10.7 ± 0.8	1:2	0.23
Two	8.1 ± 0.6	1:3	**0.02**	85.6 ± 4.2	1:3	**0.004**	3.5 ± 0.3	1:3	**0.016**	10.8 ± 0.6	1:3	**0.01**
Three	10.7 ± 0.5	2:3	0.10	94.6 ± 3.8	2:3	0.26	4.5 ± 0.2	2:3	0.162	12.9 ± 0.6	2:3	0.15
*Site*												
Beach	4.4 ± 0.6	L:B	**<0.001**	78.3 ± 6.0	L:B	0.11	1.8 ± 0.3	L:B	**<0.001**	7.28 ± 0.9	L:B	**<0.001**
Ledge	11.6 ± 0.6	N:B	**<0.001**	93.7 ± 3.7	N:B	0.92	4.9 ± 0.2	N:B	**<0.001**	13.5 ± 0.6	N:B	**0.02**
North side	9.4 ± 0.6	B:N	**0.003**	84.8 ± 4.1	B:N	0.08	4.1 ± 0.08	B:N	**0.0010**	11.1 ± 0.6	B:N	**0.005**
*Year*												
2010	8.0 ± 0.6	2010:2011	0.91	90.0 ± 5.2	2010:2011	**0.02**	3.2 ± 0.2	2010:2011	0.370	10.6 ± 0.6	2010:2011	0.76
2011	10.1 ± 0.7	2010:2012	0.38	77.5 ± 3.0	2010:2012	0.97	4.4 ± 0.3	2010:2012	0.072	12.4 ± 0.9	2010:2012	0.72
2012	10.2 ± 0.6	2011:2012	0.67	95.6 ± 4.7	2011:2012	**0.02**	4.4 ± 0.3	2011:2012	0.754	12.3 ± 0.6	2011:2012	1
	*Slope*						*Slope*			*Slope*		
Days	−0.009		**0.03**	−0.27		0.054	−0.013		0.361	−0.12		0.40
Age	−0.007		0.67	0.003		**0.049**	−0.01		0.052	−0.11		0.99

*p* values indicate significance of ANOVA test of the variables stated in the contrast column. Bold values = significant *p* values

### Trip duration

Trip duration was sensitive to the year of study with significant differences (*F* = 5.9, *df* = 2, *p* < 0.02) observed in trip duration between 2010 (mean 90 ± 5, range 13–407 min) and 2011 (mean 76 ± 3, range 14–283 min), and between 2011 and 2012 (mean 96 ± 5, range 10–439 min) (Fig. 2). Sex also significantly affected trip duration (*F* = 2.74, *df* = 1, *p* = 0.04), with males having a shorter trip duration (mean 72.5 ± 3, range 10–408 min) than females (mean 94.6 ± 5, range 13–429 min). Individuals raising one chick at the time of tracking had significantly (*F* = 5.2, *df* = 2, *p* = 0.0004) shorter trip durations (mean 75 ± 5, range 19–240 min) than those raising three chicks (mean 95 ± 4, range 13–439 min). The age of chicks also significantly increased trip duration (*F* = 8.0, *df* = 1, *p* = 0.049) (Table 3).

### Maximum distance travelled

Maximum distance travelled was also found to be significantly different between the sexes (*F* = 13.59, *df* = 2, *p* = 0.001) with females travelling further (mean 4.6 ± 0.2, range 0.3–23 km) than males (mean 3.6 ± 0.2, range 0.2–19 km) (Fig. 2). Significant differences (*F* = 4.8, *df* = 2, *p* = 0.016) were also observed in the maximum distance travelled between individuals raising one chick (mean 3.7 ± 0.3, range 0.3–10 km) compared with individuals raising three chicks (mean 4.5 ± 0.2, range 0.3–23 km) and between all breeding sites (*F* = 34.2, *df* = 2, *p* < 0.001) with shags breeding at the ledge site exhibiting the greatest maximum distance travelled (mean 4.9 ± 0.2, range 0.3–19 km) compared with those breeding at the North site (mean 4.1 ± 0.08, range 0.3–23 km) and those breeding at the beach site (mean 1.8 ± 0.3, 0.2–20 km) (Table 3).

### Area used

**Fig. 3:**
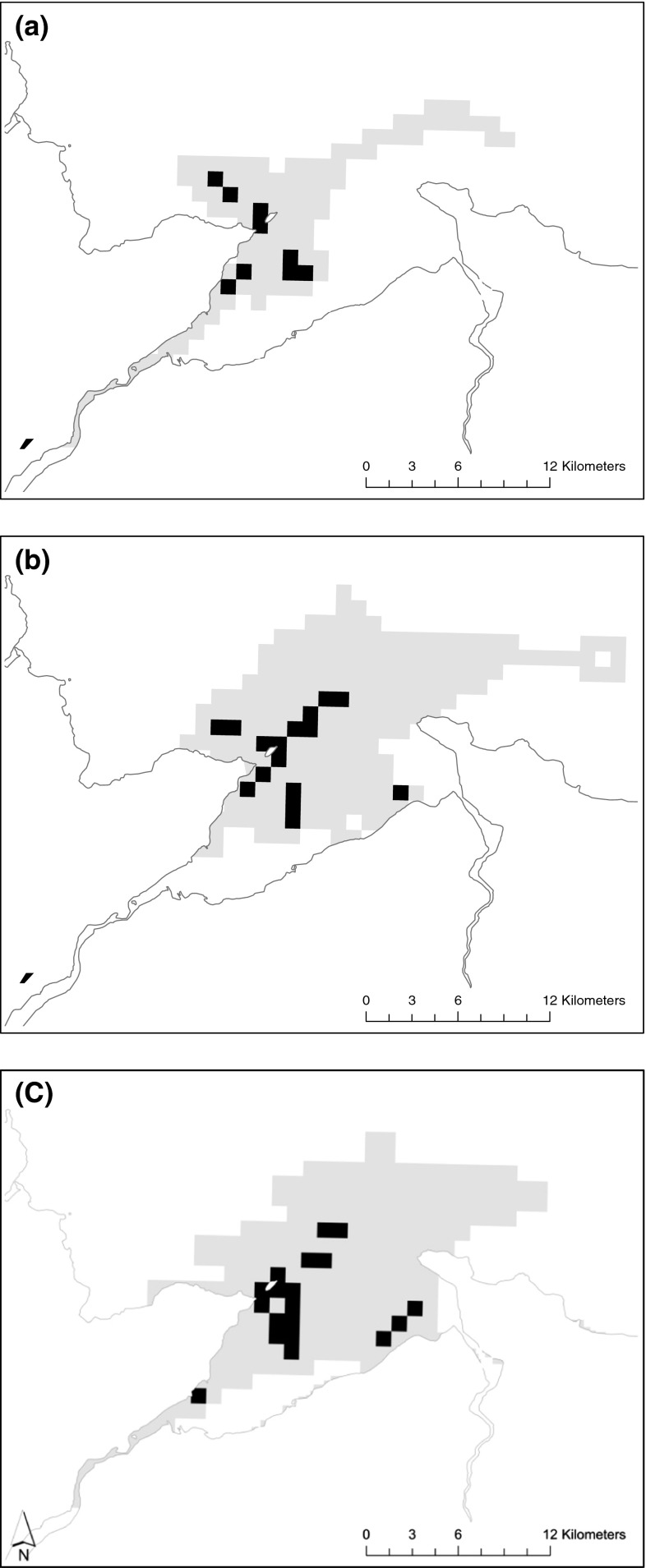
Time spent in pre-defined 1 × 1 km cells for **a** shags breeding at the beach site. **b** Shags breeding at the North site and **c** shags breeding at the ledge. *Black squares* indicate where 50 % of all time was spent; *grey squares* indicate where 100 % of time was spent

**Fig. 4:**
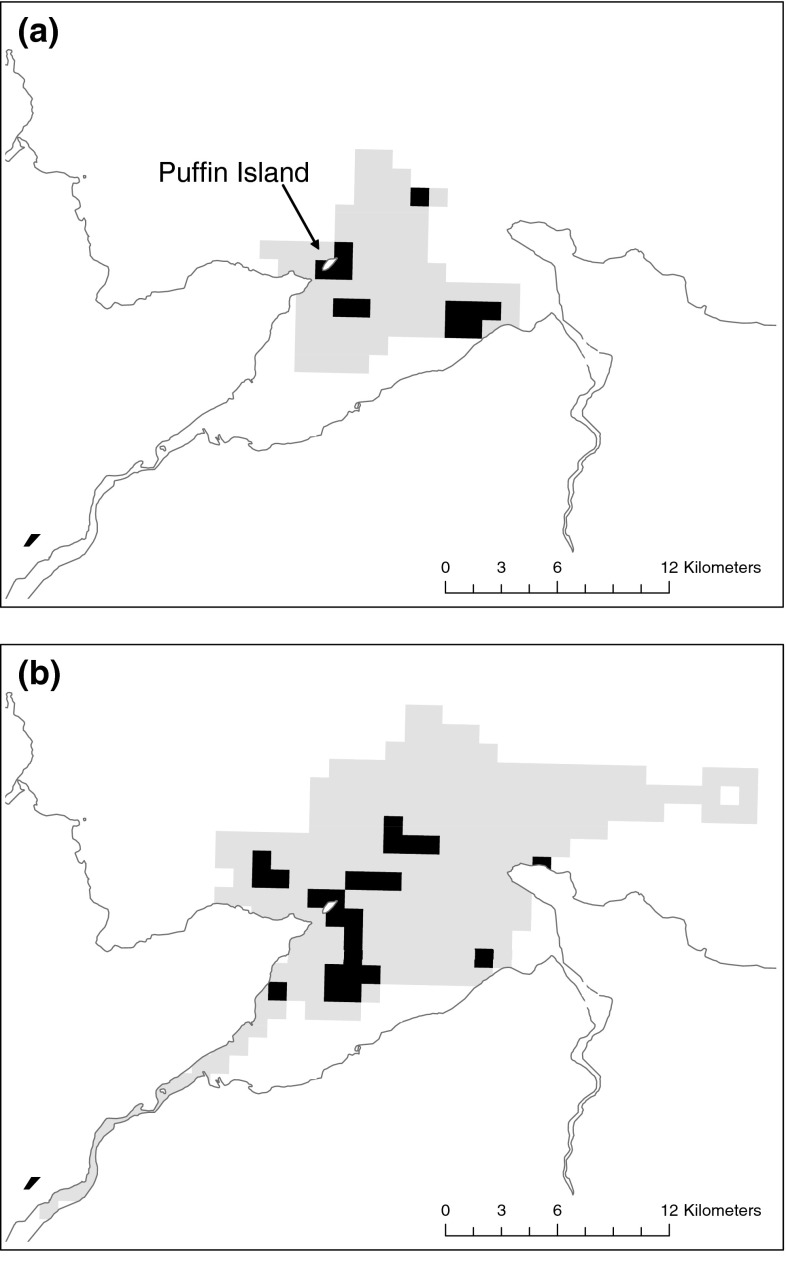
Time spent in pre-defined 1 × 1 km cells for **a** shags with one chick. **b** Shags with three chicks. *Black squares* indicate where 50 % of all time was spent; *grey squares* indicate where 100 % of time was spent

The area used (km^2^) on each foraging trip was sensitive to breeding site with significant differences found between all sites (*F* = 15.3, *df* = 2, p < 0.05) (Table 3). Those breeding at the beach site foraged over a smaller area (mean 7.3 ± 0.9, range 1–35 km^2^) compared with those breeding at the North site (mean 11.1 ± 0.6, 1–40 km^2^) and the ledge site (mean 13.5 ± 0.6, range 1–55 km^2^) (Fig. 2). Significant differences in foraging (*F* = 5.22, *df* = 2, *p* < 0.005) area were also observed between shags rearing one chick (mean 10.7 ± 0.8, range 1–27 km^2^) compared with those rearing three chicks (mean 12.9 ± 0.6, 1–49 km^2^) (Fig. 3). Maps of time spent in 1 km^2^ cells revealed different areas of use for shags rearing one chick compared with those rearing three chicks and for shags breeding at the three different sites (Figs. 3, 4).

### Direction travelled

**Fig. 5:**
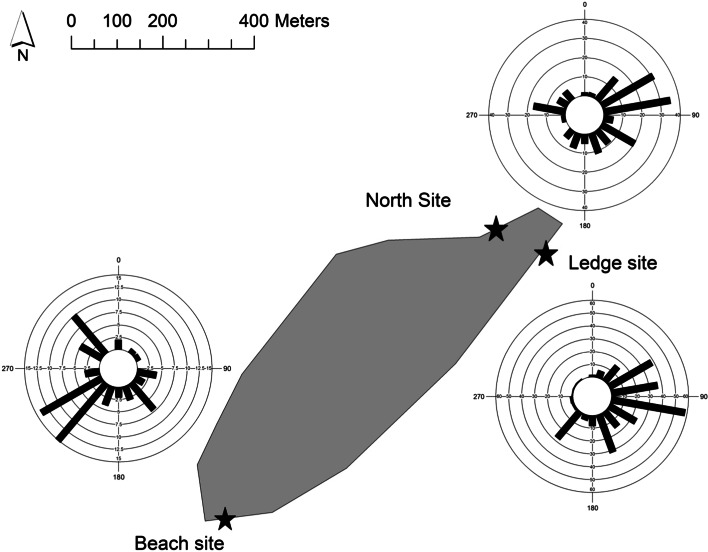
The direction travelled on each foraging trip made at the three sites on the island (beach, ledge and North breeding sites)

For direction travelled, significant differences were found between sites (*F*_2, 520_ = 78.5, *p* < 0.001). Pair-wise comparisons revealed significant differences between the beach and North site (*F*_250_ = 139.7, *p* < 0.001); beach and ledge (*F*_342_ = 140.6, *p* < 0.001) and ledge and North site (*F*_448_ = 17.0, *p* < 0.001). Trips originating from the beach site travelled a mean bearing of 22.6° (SD 73.3°), from the North side site 70.3° (SD 80.8°) and from the ledge site 100.5° (SD 53.8°) (Fig. 5). Significant differences (*p* < 0.05) were not observed in the direction travelled between males and females, between years or between individuals which were raising one, two or three chicks at the time of tracking.

### Effect of sampling regime

**Fig. 6:**
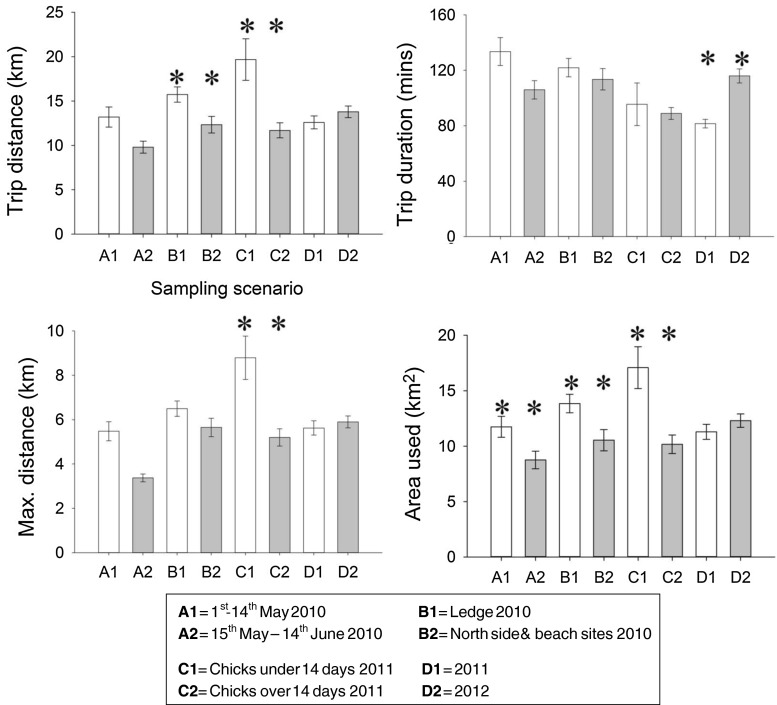
The effect of different sampling scenarios on European shag foraging trip distance, trip duration, maximum distance travelled from the colony and the area used. *Asterisks* represent significant differences between groups

Different sampling regimes produced different estimates for all foraging trip response variables (Fig. 6), in some cases predicting a difference of up to 50 % for the foraging trip parameters. Each pair of sampling regimes induced at least one significant difference in a foraging trip response variable. For example, using a sample from 2010 tracked over a 3-week period between mid May to the first week of June produced a foraging trip distance of 9.8 (SE ± 0.7) km compared with 13.2 (±1.1) km for a sample tracked in the same year but earlier in the season (Fig. 5, A1 vs. A2). Tracking individuals breeding at the ledge site in 2010 produced a foraging area estimate of 13.8 (±0.8) km^2^ compared with 10.5 (±0.95) km^2^ when sampling individuals only from the North and beach sites (Fig. 5, B1 vs. B2). Similarly, a sample tracked in 2011 with chicks under 14 days old produced a maximum foraging trip distance of 8.8 (±0.97) km compared with 5.1 (±0.38) km from a sample of individuals tracked in the same year but that was composed only of individuals with chicks over 14 days old (Fig. 5, C1 vs. C2). Trip duration was found to be greater by 26 min in 2012 (116 ± 15 min) compared with trip duration in 2011 (82 ± 14 min) (Fig. 5, D1 vs. D2).

## Discussion

This is one of the few published studies reporting the foraging behaviour of the European shag determined using GPS technology. The European shag is widespread throughout north-western and southern Europe, where more than 75 % of the global population is found. In the United Kingdom, this species breeds on coastal sites, mainly in the north and west, where over half their population is found at fewer than ten sites, making this an Amber listed species (Eaton et al. 2009). Previous studies on this species have used observations at sea and radio telemetry techniques (Wanless et al. 1991, 1998; Elkins and Williams 1974), and one other recent GPS tracking study of this species has been reported from a study site in France (Fortin et al. 2013). Understanding the foraging behaviour of the European shag is important, particularly with the anticipated rise in offshore marine renewable developments occurring in European waters (RenewableUK 2011). Given the coastal nature of this species, it is likely to be more susceptible to offshore marine developments (Langton et al. 2011).

Intraspecific variation in foraging ecology has previously been observed for other central-place foragers. For example, Austin et al. (2004) found wide variation in the foraging ecology of individual Grey seals *Halichoerus grypus*, and warned that the practice of examining average responses over populations obscures variability in behavioural ecology. Similarly, Antarctic fur seals *Arctocephalus gazella* were found to exhibit two foraging tactics which were repeatable within an individual (Bonadonna et al. 2001) and Pelagic cormorants *P. pelagicus* from the same colony were found to have distinct diving patterns (Kotzerka et al. 2011). The degree of individual specialisation varies widely within a population as a result of the diverse array of physiological, behavioural and ecological mechanisms that can act upon an individual (Bolnick et al. 2003). This study conducted over three consecutive field seasons revealed that all five of the foraging trip response variables examined can all be influenced by the explanatory factors included in the GEE model (sex, age of chicks, number of chicks, breeding site, day of season and year of study).

### Effect of breeding site

Breeding site significantly affected total trip duration, the maximum distance travelled from the colony and the area used. Those breeding at the beach site had a reduced foraging trip duration, maximum distance and area compared with those breeding at the North site and the ledge. The direction travelled between these sites was also found to be significantly different. This is an important finding as the logistics of seabird tracking work often means only sub-colonies from any site can or are sampled. Puffin Island is only 1.4 km long and 0.5 km at its widest point, but even at this relatively small colony, significant differences in the foraging parameters of shags breeding at different sub-colonies were observed. Similarly, small-scale distribution influenced the parental foraging effort of Tufted puffins *Fratercula cirrhata* breeding at two sub-colonies only 1.5 km apart on a single island based on stable isotope analysis (Hipfner et al. 2007). At a larger geographic scale, the foraging behaviour of Gentoo penguins *Pygoscelis papua* breeding at different sites within the Kerguelen archipelago was found to be more variable that the foraging behaviour observed across the rest of its southern hemisphere range (Lescroel and Bost 2005). Individuals breeding at different sites may represent birds of different status (e.g. younger, inexperienced breeders). For example, the survival of Black-legged kittiwake *Rissa tridactyla* has been reported to be greater for those nesting in the middle of a colony compared with those on the outskirts, probably related to intrinsically fit and less fit individuals (Aebischer and Coulson 1990). However, European shags have been reported as either selecting nesting sites randomly or that low-quality birds nest preferentially closer to higher quality individuals (Velando and Freire 2001). No significant differences were found in the number of chicks raised between the sites in this study. Therefore, factors other than individual quality may explain differences in the foraging behaviour at these breeding sites such as bathymetric or oceanographic features around the island (Wienecke and Robertson 2006).

### Effect of size of brood

Total foraging trip distance, trip duration, the maximum distance travelled from the colony and the area used were all significantly affected by the number of chicks an individual was raising. Those raising three chicks made significantly longer trips than those raising one chick. No previously reported studies have related foraging trip characteristics to the number of chicks a seabird is rearing. However, studies have examined parental effort in relation to brood size, and for example, the number of feeding sessions was reported to be significantly greater for Laughing gulls *Leucophaeus atricilla* rearing three chicks compared with one chick (Gonzalez-Medina et al. 2010). Similarly, Common terns *Sterna hirundo*, that generally raise three chicks, exhibited a higher rate of food delivery than Arctic terns *Sterna paradisaea*, which generally raise two chicks, (despite the adults birds being of similar size and morphology), thus indicating that having more chicks resulted in shorter foraging trips (Robinson et al. 2001). This study found individuals raising three chicks made longer foraging trips, perhaps indicating more favourable foraging grounds further from the colony (food limitation close to the colony) or that higher quality individuals had the ability to exploit these better resources and therefore provision for more chicks (Lescroel and Bost 2005; Lescroel et al. 2010).

### Effect of sex

Total trip distance, trip duration and the maximum distance travelled were also significantly affected by sex, with the foraging trips of females covering a greater distance, having a longer duration and being further from the colony than males. Differences in the foraging behaviour between sexes have been widely reported for many seabird species. For example, in Brown boobies *Sula leucogaster* and Blue-footed boobies *Sula nebouxii*, both species with reversed sexual dimorphism, females performed longer foraging trips, foraged farther from the colony, flew greater distances and had larger zones of area-restricted search than males (Weimerskirch et al. 2009). Male and female Imperial cormorants *P. atriceps* have also been reported to travel away from their colony using routes virtually perpendicular to each other so that their foraging areas were distinctly different, with females foraging close to the coast whilst males foraged offshore in deeper water (Quintana et al. 2011). These studies and the present study represent sexually dimorphic seabirds which could explain the differences observed although studies where males and females are monomorphic have also been reported. For example, female Brunnich’s guillemot *Uria lomvia* were found to forage more during twilight periods and dived shallower than males which foraged primarily during daylight hours (Paredes et al. 2008), sexual differences in the foraging habits and activities have also been reported in the Barau’s petrel *Pterodroma baraui* throughout the breeding period (Pinet et al. 2012). For the sexually dimorphic shag in our study, the smaller females tended on average to travel further and use a larger foraging area than males perhaps reflecting their ability to dive to different depths to exploit prey resources (Quillfeldt et al. 2011; Cook et al. 2007; Kato et al. 2000) or could result from competitive exclusion (Phillips et al. 2011).

### Effect of year of study

The year of study influenced the trip duration but not the other response variables. When comparing 2011 with 2010 and 2012, birds in 2010 travelled similar distances from the colony and the same total distance as the other years but spent longer on foraging trips. The effect of year of study on foraging strategy is predictable given the variability in climatic and weather patterns in any year, which directly relate to sea surface temperature and chlorophyll a abundance which will in turn affect the productivity of the ocean (Fortin et al. 2013). Inter- annual variance in the foraging behaviour of seabirds has often been reported (Chivers et al. 2012; Garthe et al. 2011). However, only 2 out of the 22 studies published between November 2011–November 2012 (Table 1) tracked seabirds for more than one breeding season. Whilst this may not be necessary for the objectives of some studies, for those aiming to identify important foraging ranges and foraging areas, it should be important to consider inter-annual variation given the range of environmental factors that could potentially influence the year of study.

### Effect of sampling regimes

The different hypothetical sampling regimes analysed in this study produced quite different estimates for the foraging trip variables. This study highlights the problem of failing to consider the effects of behavioural, environmental and ecological effects on an individual’s foraging behaviour. Drawing inferences to the population as a whole from samples representing a limited spatial, temporal or behavioural scale are unlikely to fully represent the population (Lindberg and Walker 2007). Whilst previous studies have examined the effects of sample size and sample composition (Morrison 1984; Blundell et al. 2001), few published studies have focused on the composition of samples when examining home-range areas of central-place foragers such as seabirds. Our analysis leads us to recommend that researchers wishing to most accurately identify or delineate home-range areas should ensure that tracking work is conducted throughout the breeding season, between years, and includes both males and females from locations evenly distributed over the field site (rather than concentrating effort in any one area).

Including a larger number of individuals and foraging trips in a sample in any single year will help reduce the influence of variability in foraging trip characteristics caused by factors such as sex, breeding site etc. (Soanes et al. 2013). Indeed, it is this variability that most likely underpins the previously reported relationship between sample size and foraging area size (Soanes et al. 2013). However, as highlighted, samples used in tracking studies are often small. The European shag is a localised coastal feeder, yet even for this relatively short distance forager, the impacts of the explanatory variables were significant on foraging response variable predictions. It is likely that seabirds which have larger foraging radii may exhibit even greater differences in their foraging behaviour in relation to the explanatory variables tested. Therefore, the selection of individuals and timing of tracking for inclusion in tracking studies of any central-place forager are important factors to consider to ensure that the limited samples often used in such studies most accurately predict the colony’s foraging characteristics.
